# Comparison of sister species identifies factors underpinning plastid compatibility in green sea slugs

**DOI:** 10.1098/rspb.2014.2519

**Published:** 2015-03-07

**Authors:** Jan de Vries, Christian Woehle, Gregor Christa, Heike Wägele, Aloysius G. M. Tielens, Peter Jahns, Sven B. Gould

**Affiliations:** 1 Institute of Molecular Evolution, Heinrich-Heine-University Düsseldorf, Universitätsstrasse 1, 40225 Düsseldorf, Germany; 2 Zoologisches Forschungsmuseum Alexander Koenig, Adenauerallee 160, 53113 Bonn, Germany; 3 Department of Biochemistry and Cell Biology, Faculty of Veterinary Medicine, Utrecht University, Utrecht, The Netherlands; 4 Department of Medical Microbiology and Infectious Diseases, Erasmus University Medical Center, Rotterdam, The Netherlands; 5 Plant Biochemistry and Stress Physiology, Heinrich-Heine-University Düsseldorf, 40225 Düsseldorf, Germany

**Keywords:** invertebrates, sacoglossa, kleptoplasty, reactive oxygen species, starvation, photosynthetic slugs

## Abstract

The only animal cells known that can maintain functional plastids (kleptoplasts) in their cytosol occur in the digestive gland epithelia of sacoglossan slugs. Only a few species of the many hundred known can profit from kleptoplasty during starvation long-term, but why is not understood. The two sister taxa *Elysia cornigera* and *Elysia timida* sequester plastids from the same algal species, but with a very different outcome: while *E. cornigera* usually dies within the first two weeks when deprived of food, *E. timida* can survive for many months to come. Here we compare the responses of the two slugs to starvation, blocked photosynthesis and light stress. The two species respond differently, but in both starvation is the main denominator that alters global gene expression profiles. The kleptoplasts' ability to fix CO_2_ decreases at a similar rate in both slugs during starvation, but only *E. cornigera* individuals die in the presence of functional kleptoplasts, concomitant with the accumulation of reactive oxygen species (ROS) in the digestive tract. We show that profiting from the acquisition of robust plastids, and key to *E. timida*'s longer survival, is determined by an increased starvation tolerance that keeps ROS levels at bay.

## 1. Introduction

Some sacoglossan slugs can house functional plastids for months in the cytosol of cells that line the animals' digestive tubules. The slugs steal the plastids (kleptoplasts) from siphonaceous algae upon which they feed. Theory has it that the presence of functional kleptoplasts [1,2] allows some of the sea slug species to survive starvation periods that can last almost a year [3,4]. Recent reports question the categorical importance of photosynthesis during starvation for several species [5,6] and while the animals fix CO_2_ in a light-dependent manner [5,7–10], for how long and how much during starvation is not well documented. Animals induce autophagy to reallocate resources through the recycling of tissue when facing starvation [11] and mitochondria-generated reactive oxygen species (ROS) signalling has been found to be a key mediator of autophagy triggered by nutrient deprivation [12,13]. Over the past 20 years, research on ‘photosynthetic (green) slugs’ has focused on understanding how the stolen plastids can remain functional in a foreign cytosol [14], but the molecular response to starvation has not, to our knowledge, been assessed in sacoglossan slugs until now.

The kleptoplasts' stability outside of the algae disagrees with our knowledge about the sensitivity of land plant plastids, whose photosystem II is highly susceptible to photosynthesis-associated damage [2,15,16]. Recent works [16,17] are reviving old observations that demonstrated some algal plastids are naturally more robust than land plant plastids [18]. Kleptoplast longevity therefore appears an intrinsic property the stolen organelles bring along, and depending on the slug species this results in different modes of kleptoplast retention and lengths of survival during starvation [2,16]. It is generally suspicious that among 300 sacoglossan species that feed on siphonaceous algae, only seven species are known to maintain functional kleptoplasts and survive long term [2,19,20].

The two congeneric species *Elysia cornigera* and *Elysia timida* both feed on the ulvophycean alga *Acetabularia*, but only *E. timida* tolerates prolonged starvation [2,21]. Why? We compared the two sister taxa by monitoring their kleptoplasts' photosynthetic capacity and characterizing the slugs' physiological response to starvation focusing on gene expression modulation and ROS development. Our results indicate that algal cytosol are the slug's main food source and furthermore demonstrate that the plastid-bearing slugs' capacity to endure prolonged periods of starvation is not determined by the photosynthetic activity of their kleptoplasts, but by how they have evolved to respond to starvation, in particular the ability to cope with ROS.

## 2. Results and discussion

### (a) Performance of stolen plastids does not depend on the slug species

**Figure 1.**
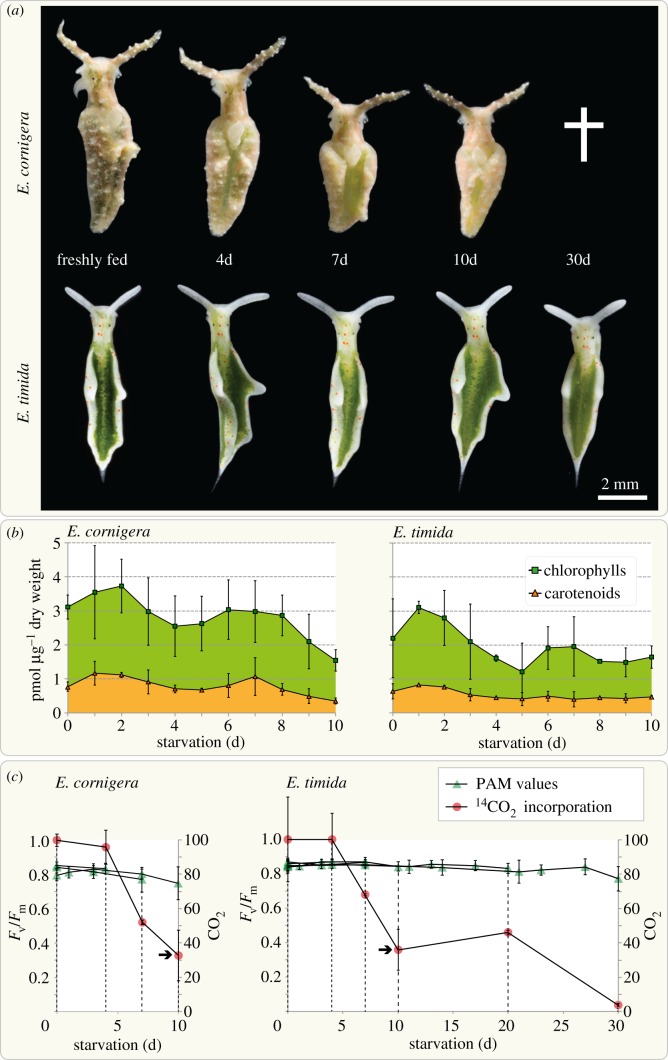
*Elysia cornigera* dies while showing comparable photosynthetic activity to *E. timida* during starvation. Adult slugs were fed on *Acetabularia acetabulum* and the effects of starvation at 25 µmol quanta m^−2^ s^−1^ on the photosynthetic capacity of both species evaluated. (*a*) Representative images of one single specimen of each species. *Elysia cornigera* shrinks more rapidly than *E. timida*, and while the *E. cornigera* specimens never survived starvation for more than two weeks, all *E. timida* specimens survived and retained an almost equal body length during the 30 d of starvation analysed. (*b*) Analysis of pigment concentration in relation to dry weight of the slugs during the first 10 days of starvation. Quantification was performed on three biological replicates per time point and normalized to an external standard; each pigment extract was derived from three to seven *E. cornigera* (in total 143 individuals) and two *E. timida* individuals (in total 66 individuals). (*c*) Assessment of the maximum quantum yield (*F*_v_/*F*_m_; measured using a PAM fluorometer) of photosystem II and net ^14^CO_2_ fixation relative to freshly fed animals (t_0_), during starvation within the same group of individuals. Each single *F*_v_/*F*_m_ curve represents measurements on a group of at least 12 *E. cornigera* and nine *E. timida* specimens; from each group four *E. cornigera* (in total 48 individuals) and three *E. timida* (in total 54 individuals) were used for determining the ^14^CO_2_ incorporation after 0, 4, 7, 10, 20 and 30 days of starvation (latter two only for *E. timida*).

We fed *E. timida* and *E. cornigera* solely on *Acetabularia acetabulum* DI1 from the day they hatched in our laboratory*.* After eight and six weeks, respectively, and at which time the animals had reached maturity, the slugs were separated from the alga and routinely kept at 25 µmol quanta m^−2^ s^−1^ for 12 h every day. All sacoglossans that starve are observed to shrink [10,22] and the same was true for our two species. Yet, *E. cornigera* decreased in size more rapidly than *E. timida* (figure 1*a*) and rarely survived more than two weeks without food, which is consistent with previous reports [19,21]. Loss in size was accompanied by a slow fading of the animals' greenish coloration owing to the loss of the pigments chlorophyll (Chl) *b* and, most prominently, Chl *a* (electronic supplementary material, table S1). In *E. cornigera*, pigment concentration declined from starvation day 2 to 4 by 32.9%, but then increased again for a few days in relation to the dry weight of the animals (figure 1*b*). At day 10, the Chl *a* and *b* concentration had dropped from 3109 to 1544 pmol mg^−1^ animal dry weight (49.7%). These values correlate with the rapid decrease observed in body size between day 4 and 7 of starvation (figure 1*a*). In *E. timida*, we observed a similar, yet less steep, decline in the concentration of pigments in relation to body mass over the 10 days of starvation measured (from 2193 to 1606 at day 4 (73.2%) and 1638 pmol Chl *a* + *b*/mg animal dry weight at day 10 (74.7%); figure 1*b*). The increase of photosynthesis-associated pigments around day 6 demonstrates that the animals we measured metabolize their own tissue at an apparently faster rate than the kleptoplasts degrade, which was also observed for starving juveniles of *E. chlorotica* [23].

Photosynthetic capacity was determined through measurements of photosystem II quantum efficiency (derived from Chl fluorescence analyses determined by pulse-amplitude modulation (PAM) fluorometry) and the amount of incorporated ^14^CO_2_. Freshly fed slugs showed a similar initial maximum quantum yield (*F*_v_/*F*_m_ of 0.83 ± 0.03 for *E. cornigera* and 0.85 ± 0.03 for *E. timida*). Measured *F*_v_/*F*_m_ of *E. timida* declined slowly (−0.001 *F*_v_/*F*_m_ per day starved) over the one-month period, reaching 0.77 ± 0.07 at day 30. In *E. cornigera*, the decline of *F*_v_/*F*_m_ was more prominent (−0.008 *F*_v_/*F*_m_ per day starved) over the 10 days measured, dropping to 0.75 ± 0.1, at which point the species began to die (figure 1*c*). At the same time, the amount of ^14^CO_2_ incorporation had dropped to 34% (±15%) and 36% (±12%) in *E. cornigera* and *E. timida*, respectively, after 10 days of starvation (figure 1*c*). At that point, the *F*_v_/*F*_m_ had dropped to 0.84 ± 0.03 and 0.75 ± 0.1 for *E. timida* and *E. cornigera*, respectively. After 30 days of starvation, the amount of ^14^CO_2_ fixed in *E. timida* had dropped to 4 ± 1% in regard to the initial amount. In any case, after 10 days of food deprivation *E. cornigera* died in the presence of intact (electronic supplementary material, figure S1) and functional kleptoplast. The difference of profiting from kleptoplasty must depend on the animals' tolerance to starvation, not the stolen organelles' performance. This observation challenges the idea that kleptoplasts could act as food depot [5,10], and raised the question, why in particular *E. cornigera* dies in the presence of functional kleptoplasts.

### (b) Global gene expression response is predominantly governed by starvation

To uncover the differences of the two species in the response to starvation, and test whether the slugs can to some respect sense the kleptoplasts' status, we analysed gene expression changes throughout starvation and under different environmental stimuli. We performed comparative transcriptomics of the slugs: (i) under starvation (S), (ii) under starvation and in the presence of the photosynthesis inhibitor drug monolinuron (S + M), and (iii) under starvation and including a daily bleaching pulse with 1000 µmol quanta m^−2^ s^−1^ for 1 h d^−1^ (S + B). A total of 10 857 contigs for *E. cornigera* and 11 152 contigs for *E. timida* were assembled that were supported by at least 100 reads and homologous to eumetazoan sequences (electronic supplementary material, figure S2 and table S2). Global gene expression trends were confirmed for all conditions on six individual genes using quantitative reverse-transcription PCR (qRT-PCR) (electronic supplementary material, figure S3).

**Figure 2.**
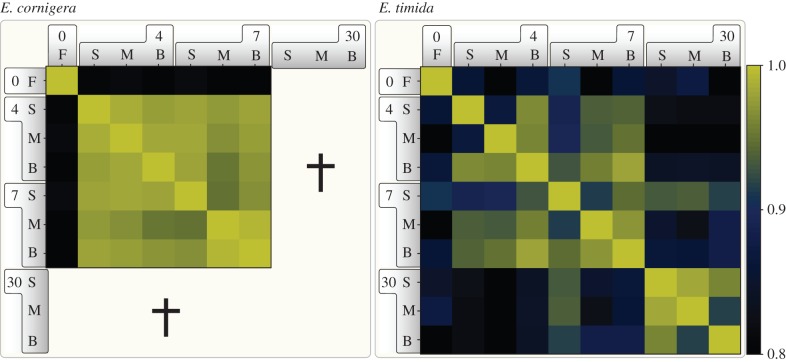
Global co-expression analysis of gene expression changes. Pearson correlations were calculated by comparing the FPKM (fragments per kilobase of exon per million reads mapped) values of filtered genes of the different samples. Note the clear separation of the control and the remaining conditions and for *E. timida* the shift between the time points of 4 and 7 days in comparison to 30 days. The colour key indicates the degree of co-regulation. The bold numbers represent time of treatment in days. No values for *E. cornigera* were obtained for 30 days, because all individuals had succumbed to starvation (indicated by a cross). F, freshly fed; S, starving; M, monolinuron; B, bleach.

If the slugs have evolved to depend on the photosynthetic capacity of their kleptoplasts, we would expect to see a strong difference in the regulation of global gene expression profiles when kleptoplast electron transport is blocked (S + M) or when the kleptoplasts are under high light stress (S + B). Yet, when analysing the co-regulation of gene expression we found that starvation (S) in comparison to feeding (F) was the main denominator for the transcription response in *E. cornigera* (figure 2). The additionally applied treatments (S + B, S + M) only had marginal effects on the global expression profile of *E. cornigera* compared to starvation alone (S). In contrast to this, the response of *E. timida* throughout the first two weeks of starvation is more differentiated in regard to the individual stimuli and in comparison to *E. cornigera* (figure 2). After 4 d of S + M and 7 d S + B, the most differing expression was detected, indicating that *E. timida* indeed responded to the status of their kleptoplasts. Intriguingly, for *E. timida* a similar yet delayed transcriptional response to starvation was observed, as transcriptional profiles at day 30 began to reflect those observed for *E. cornigera* earlier during starvation based on the correlation of co-regulated genes within each species (figure 2). The slugs predominantly respond to starvation and not to the photosynthetic capacity of the kleptoplasts.

### (c) Host response underpins kleptoplast compatibility

**Figure 3.**
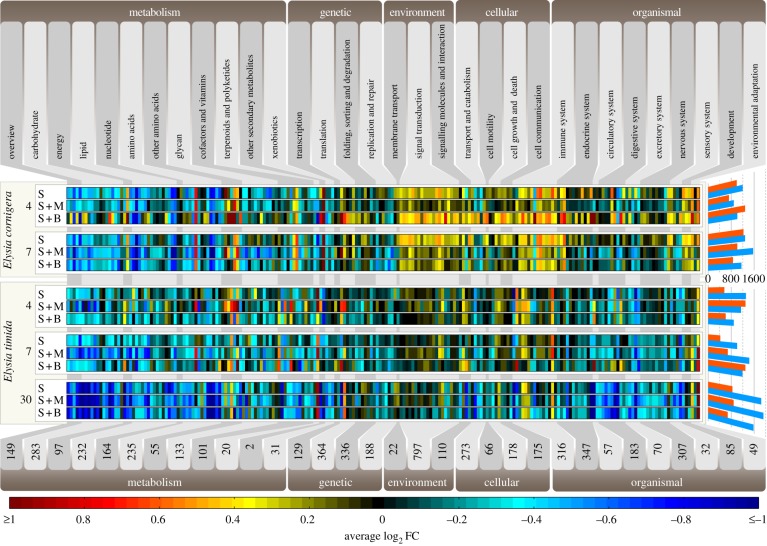
Gene expression changes of animal KEGG pathways. Transcriptomes were sequenced for both animals after 4 and 7 days of starvation (bold numbers), and additionally after 30 days for *E. timida. Elysia cornigera* and *E. timida* were subjected to three different conditions: starvation alone (S), starvation plus monolinuron (S + M; 2 µg ml^−1^) and starvation plus bleaching (S + B; 1000 µmol quanta m^−2^ s^−1^ for 1 h each day). Upregulation is shown from red to black and downregulation from black to blue. The numbers at the bottom represents the amount of K-numbers (KEGG orthology group identifier) found within each reaction module (modules in alternating light and grey flags). The bars on the right show the total number of regulated genes (minimum of twofold) for the individual conditions analysed (blue bars, downregulation; red bars, upregulation).

When mapping the transcriptomes to the Kyoto Encyclopedia of Genes and Genomes (KEGG) database (figure 3), two main trends were observed: first, downregulation of carbohydrate, energy, and lipid metabolism associated pathways (mean log_2_FC of −0.32 versus an overall mean log_2_FC of −0.10). On average, 81 of the 89 metabolism pathways detected for *E. timida* were downregulated after 30 days of starvation. Monolinuron, blocking the quinon-binding site of the D1 protein [24], enforced the downregulation of core metabolic genes in both species (at 7 d S + M log_2_FC −0.44 in both). Second, in contrast to *E. timida* (mean log_2_FC of −0.11), starvation in *E. cornigera* triggered an elevated expression of environmental information processes and cellular and organismal systems including stress response and autophagy (mean log_2_FC 0.13). Light stress further promoted this reaction in *E. cornigera* (mean log_2_FC of 0.20 at 4 d/7 d S + B) compared to *E. timida* (mean log_2_FC of –0.03 at 4d/7d S + B). Starvation imposes a metabolic dormancy and gene expression profiles to a minor degree reflect kleptoplast manipulation. The activity of the kleptoplasts, however, does not explain the survival during starvation.

Ubiquitination is a key mechanism to tag organelles and misfolded proteins—accumulating under stress conditions such as starvation—for degradation [25,26]. Expression levels of polyubiquitin remained the same in both species, except for a single polyubiquitin homolog of *E. timida* (Eti000028, 4.5-fold upregulation, *p* < 0.05). For the ubiquitin binding autophagic receptor p62/SQSTM1 [27], an average of only 28 FPKMs (fragments per kilobase of exon per million reads mapped) were identified for *E. cornigera*, but 659 FPKMs for *E. timida*. And only for the latter species an upregulation of *p62/SQSTM1* was observed during starvation (electronic supplementary material, table S3). *Elysia timida* might be better equipped to detect damaged proteins and organelles than *E. cornigera*, allowing the former to better recognize and remove damaged organelles and misfolded hazardous proteins during starvation. The ability to recognize damaged kleptoplasts for their subsequent degradation could explain how the slugs profit from stored kleptoplasts. Only malfunctioning and ROS-leaking plastids would be detected by the autophagosomal machinery and removed; the profit of kleptoplasts would be an indirect one. This would also explain the more rapid decline of *F*_v_/*F*_m_ values in *E. cornigera* in comparison to *E. timida*, while retaining the same CO_2_ fixation rate (figure 1*c*). Accumulation of damaged plastids that fix no CO_2_ would lead to a lower *F*_v_/*F*_m_ ratio in *E. cornigera*. If *E. timida* specifically removes damaged plastids, then all malfunctioning photosystems are excluded from that equation.

We found that the upregulation of genes encoding ROS scavengers differed between the two sister taxa. With progressing starvation, *E. cornigera* elevated the expression of superoxide-dismutase homologs regardless of the treatment (e.g. Eco000152; on average 3.1-fold up at day 7, *p* < 0.05). Apparent changes in the expression of catalases were only detected for *E. timida* in the first days of starvation (Eti001444; up more than twofold on average). It was recently noted that a crucial part of the slug-plastid symbiosis is evolving mechanisms to cope with kleptoplast-associated toxins [2]. A difference in stress response and ROS evolution could explain the different survival rates of the two species observed during starvation.

### (d) *Elysia cornigera* accumulates reactive oxygen species, not *Elysia timida*

**Figure 4.**
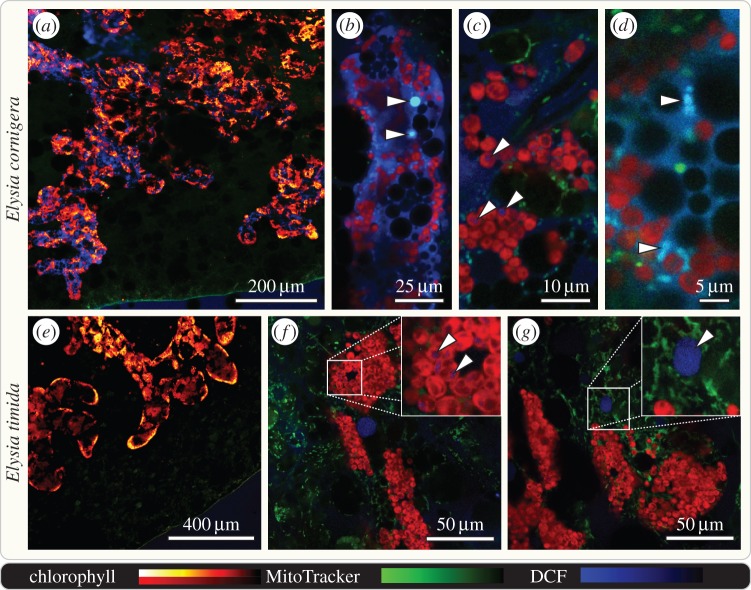
Hydrogen peroxide accumulates in the digestive tubules of starving *E. cornigera*, but not *E. timida*. Confocal laser scanning micrographs of the kleptoplast-bearing digestive tubules that pervade the parapodia of 10 days starved, whole mount *E. cornigera* and *E. timida* stained with 100 µM dichlorofluorescin (DCF; blue) and MitoTracker Red (green); chlorophyll autofluorescence in red to yellow (*a*,*e*) or red only (*b*–*d*, *f*, *g*). Digestive tubules of *E. cornigera* (lined with autofluorescent kleptoplasts) show well-defined DCF staining within the lumen and epithelium (*a*–*d*). Details of single digestive tubules revealing readily H_2_O_2_ accumulators of equal size as kleptoplasts (*b* arrowheads), kleptoplasts displaying weak DCF staining (*c*; see arrowheads) and MitoTracker Red co-localizing with DCF staining (*d*). Digestive tubules of *E. timida* displaying no distinguishable DCF signal in epithelium or lumen (*e*). Details of single digestive tubules revealing accumulating weak levels of H_2_O_2_ (*f*, arrowheads), comparable to those seen in *E. cornigera* (*c*, arrowheads) and showing globular structures of unknown nature accumulating H_2_O_2_ (*g*, arrowhead).

**Figure 5.**
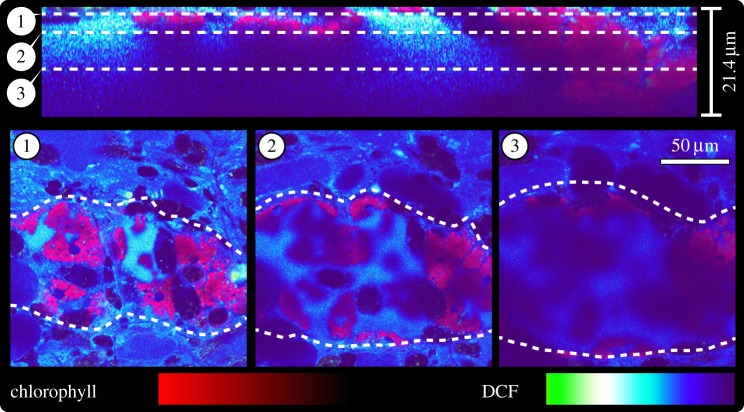
Hydrogen peroxide is present in the lumen and epithelium of digestive tubules of starving *E. cornigera*. Orthogonal view of a confocal laser scanning *z*-stack micrograph reconstructed from 58 individual optical sections (21.35 µm of depth in total) of a single digestive tubule (outlined by dashed lines in the bottom panel) of 10d starved *E. cornigera* stained with 100 µM dichlorofluorescin (DCF; purple-blue to green gradient); chlorophyll autofluorescence in red. At the top, a transverse section trough the stack is shown to illustrate at which level the three coronal sections (indicated by dashed lines) were captured.

ROS are known as signalling molecules [13] but also as deleterious cytotoxins [28]. To monitor hydrogen peroxide (H_2_O_2_) production on tissue level we used 2′,7′-dichlorofluorescin (DCF). Freshly fed slugs showed no detectable amount of H_2_O_2_ (electronic supplementary material, figure S4), but at day 10 of starvation H_2_O_2_ was most prominently detected in *E. cornigera* within the lumen of the digestive tubules (figure 4*a*,*e*). Both the lumen and the epithelium of the digestive tubules—the localization of the kleptoplasts—showed intensive DCF staining (figure 5). By contrast, DCF staining in *E. timida* occurred only in some globular structures of 10–20 µm in diameter, but of otherwise unknown nature, within individual digestive tubules (figure 4*g*, arrowhead). After 30 d of starvation, the digestive glandular tubules of *E. timida* still showed no apparent increase in H_2_O_2_ levels (electronic supplementary material, figure S4), but DCF now additionally stained globular structures of approximately 100 µm in diameter that were situated between the epidermis and the digestive tubules, and independent from the latter. These structures could represent one of the secondary metabolite-harbouring vacuoles typical for these kind of slugs [29]. Stroma of the *A. acetabulum* kleptoplasts resting within the digestives tubule cells of both *E. cornigera* and *E. timida* showed equal intensity in regard to H_2_O_2_ levels (figure 4*c*, *f* insert). Mitochondria, a common source of ROS [13], were found to accumulate H_2_O_2_ as proved through the co-localization of the DCF signal with MitoTracker Red (figure 4*d*). Hydrogen peroxide, a critical ROS in terms of cytotoxicity [28], accumulates only in *E. cornigera*.

**Figure 6.**
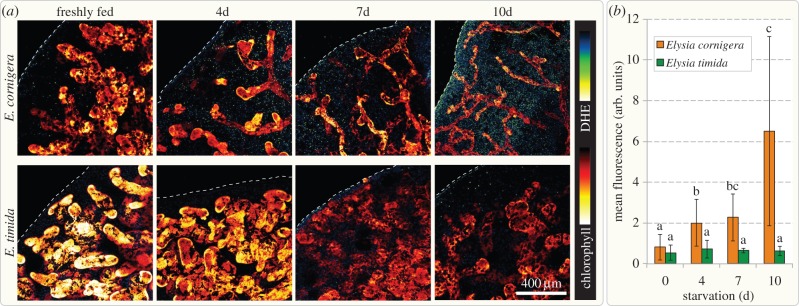
Superoxide accumulation in starving slugs. (*a*) Confocal laser scanning micrographs of the kleptoplast-bearing digestive tubules from similar positions on the parapodia of freshly fed and 4, 7 and 10 days starved, whole mount *E. cornigera* and *E. timida* (optical sections; pinhole set to 1 airy unit) stained with 100 µM DHE (staining 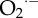), shown in blue-yellow gradient; chlorophyll autofluorescence of the kleptoplasts is shown as red to yellow glow. Dashed lines mark the outer rim of the parapodia. DHE signal increases over time in *E. cornigera*, while the diameter of the digestive tubules decreases, visible due to their lining with kleptoplasts. Superoxide in the parapodia of *E. timida* does not significantly increase over time, while kleptoplasts lining the digestive tubules appear less densely packed. (*b*) Quantification of the mean DHE fluorescence intensity in the parapodial tissue; significance groups (for (*a,b*) *p* < 0.01; (*c*) *p* < 0.05) were determined using a Mann–Whitney *U*-test; measurement of fluorescence intensity was performed on greater than or equal to six individual specimens for each time point; error bars indicate standard deviation.

Animals respond to nutrient depletion by autophagy, and superoxide (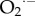) is a key signal that triggers the autophagosomal machinery [12,13] and mammalian tissues cultures show strong increase in ROS after a few hours of starvation [12]. We conducted nine separate experiments, each time with different individuals, and monitored 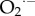 evolution using dihydroethidium (DHE) which stains the nuclei of cells that experience elevated levels of 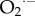 [30]. Chl autofluorescence was observed to decrease, but not completely fade, in both species throughout the 10 d monitored (figure 6*a*). This concurs with the pigment measurements and the ability of the remaining kleptoplasts to incorporate ^14^CO_2_ (figure 1*c*). Feeding slugs showed only faint 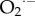 signals towards the edges of the parapodia, especially the epidermis (figure 6*a*). Staining did not predominantly dye nuclei of the plastid-bearing digestive gland cells, but instead those of surrounding and connective tissue of the parapodia. Levels of 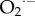, apparent through the number of nuclei stained and the intensity of the staining itself, steadily increased throughout starvation and predominantly in *E. cornigera* (figure 6*a*). While both slug species harboured functional kleptoplasts stemming from the same source, only *E. cornigera* exhibited increasing levels of 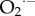 that were significant, but not *E. timida* (*p* < 0.001). *Elysia cornigera* accumulates ROS to a much higher degree than its sister taxon (figure 6*b*), reflecting the different capacities of the two species with regard to managing starvation in the presence of functional kleptoplasts.

## 3. Conclusion

Contrary to the current view our study demonstrates that the presence of functional kleptoplasts alone does not underpin the survival of green photosynthetic slugs. It is first and foremost the ability of some species to tolerate starvation. The best hints we currently have that underpin starvation tolerance include the upregulation of a set of canonical ROS quenchers such as catalases, and/or slug-specific polyproprionate metabolites such as Elysiapyrone or Elysione [31], and/or the efficient and specific degradation of damaged kleptoplasts. Literature has divided slugs into non-, short- and long-term retention species, depending on for how long kleptoplasts are functionally retained [19]. Our results demonstrate that it is equally important to divide the slugs into starvation-intolerant and starvation-tolerant species. That tolerance might be associated with the habitat and availability of the food source. *Elysia cornigera* was collected from the Caribbean Sea, but *E. timida* from the Mediterranean. Maybe only *E. timida* experiences food shortage due to seasonal differences and a difference in the calcification of individual algal species, and had to evolve accordingly. In any case, sacoglossan research has focused on how the animals chaperone their kleptoplasts, but it should just as much focus on how they manage starvation, because it will teach us on how they suppress ROS stress.

## 4. Experimental procedures

*Elysia timida* was collected on Giglio, Italy (42°22′ N, 10°52′ E and 42°21′ N, 10°52′ E) between 3 and 6 m depth. *Elysia cornigera* was collected at the Florida Keys (24°38′ N, 81°18′ W) at about 1 m depth. Both species were kept at a 12 L : 12 D rhythm at 25 µmol quanta m^−2^ s^−1^ in 3.7% salt water (Tropic Marine) and at 21°C. *Acetabularia acetabulum* DI1 (isolate of Professor Diedrik Menzel, Bonn University, Germany) was grown at the same conditions as the animals, but in algal f/2 medium. For starvation experiments, animals were moved to Petri dishes (∅︀200 mm) and 50% of the water was changed every other day. Monolinuron (JBL GmbH) was added from a stock solution of 4000 mg l^−1^ to a final concentration of 2 µg ml^−1^. Bleaching was performed by illuminating the slugs with 1000 µmol quanta m^−2^ s^−1^ for 1 h every day at the same time. For each pigment extraction, pooled slugs were transferred to 90% acetone for each time point, homogenized and kept at −20°C for 1 day. Extractions for every time point were carried out as biological triplicates, each replicate consisting of at least three *E. cornigera* and two *E. timida*. Crude extracts were filtered through a 200 nM polytetrafluoroethylene membrane and then analysed by reversed-phases high pressure liquid chromatography with ultraviolet/visible spectroscopy detection (Hitachi/Merk) as described earlier [32]. Pigment concentrations were determined using external pigment standards isolated from spinach thylakoids. Dry weight was determined on dried slug homogenate after extraction.

Photosystem II maximum quantum yield (*F*_v_/*F*_m_) was determined using a WALZ MINI-PAM as described previously [33], on the same group of individuals that were used for ^14^CO_2_ incorporation measurements. For every time point for which ^14^CO_2_ incorporation was determined, an individual group of slugs (at least 12 *E. cornigera* or nine *E. timida*) was kept in isolated dishes. For each of these groups, separate *F*_v_/*F*_m_ measurements were performed, and each individual slug of a group was measured at least three times. For each data point, the mean of all individual triplicate measurements was calculated. The light-driven incorporation of ^14^CO_2_ was determined as described earlier [5]. Briefly, for each triplicate measurement four individuals of *E. cornigera* and three of *E. timida* were used. Slugs were incubated in 1.2 ml artificial seawater supplemented with 0.40 mM [^14^C]-NaHCO_2_ (25 µCi per incubation, NEN-radiochemicals, MA, USA) for 2 h at room temperature and 72 µmol quanta m^−2^ s^−1^ illumination. After rinsing, homogenization and acidification of the material with 150 µl 1 M HCl, all the substrate was expelled from the homogenate. Incorporation of ^14^C by the slugs was determined in a scintillation counter after the addition of 12 ml LUMA-Gel scintillation cocktail (LUMAC, The Netherlands).

For ROS-imaging, slugs were incubated for 45 min with 100 µM DHE (DHE; excitation/emission HeNe 543/610 nm; Sigma) or 100 µM DCF (2′,7′-dichlorofluorescin; excitation/emission Argon 488/529 nm) plus 2 µM MitoTracker Red CMXRos (excitation/emission HeNe 543/599 nm; LifeTechnologies) in artificial seawater. Slugs were rinsed twice with seawater and decapitated before mounting. Confocal laser scanning microscopy was carried out using a Zeiss LSM 710. Imaging was always performed with the same settings and at a similar position along the parapodial rim at the same depth relative to the epidermis. Images were processed using Fiji/ImageJ 1.48f [34] and statistics were performed using R [35]. Normality was tested via a Shapiro–Wilk test [36] and significance using a Mann–Whitney *U*-test [37].

Total RNA was extracted three times from seven *E. cornigera* (a total of 21) and three times from five *E. timida* (a total of 15) feeding on *A. acetabulum* (*t*_0_, feeding control group), 28 individuals of *E. cornigera* that were starved for 4 days, of which nine individuals had only starved (S), nine had starved and were treated with monolinuron (S + M), and 10 of which had starved and were treated with 1000 µmol quanta m^−2^ s^−1^ for 1 h each day (S + B). The RNA of 20 *E. timida*'s (7 S, 7 S + M, 6 S + B) was isolated after four days of starvation (*t*_1_), of 30 *E. cornigera* (10 S, 9 S + M, 11 S + B) and of 20 *E. timida* (6 S, 8 S + M, 6 S + B) after 7 days of starvation (*t*_2_), and finally of 32 *E. timida* (11 S, 10 S + M, 11 S + B) after 30 days of starvation (*t*_3_). RNA was isolated using TRIzol (Life Technologies) according to the manufacturer's protocol and an additional DNAse treatment (Thermo Fisher Scientific). Poly(A) mRNA enrichment, library preparation using the TruSeq kit (Illumina) and 100 bp paired end sequencing using the Illumina HiSeq2000 system was performed by the Beijing Genome Institute (Hong Kong). Reads with remaining adapter sequences, more than 5% of unknown nucleotides or more than 20% of nucleotides with quality scores less than 10 (Illumina 1.5) were removed as part of the raw data extraction.

A total of 1 186 405 486 reads (a minimum of 52 million reads/library) were obtained (electronic supplementary material, table S2). Reads were inspected by FastQC v. 0.10.1 [38] and filtered and trimmed using Trimmomatic 0.32 [39] (parameters: ILLUMINACLIP:TruSeq3-PE.fa:2 : 30 : 10; HEADCROP:10; TRAILING:3; SLIDINGWINDOW:4 : 20; MINLEN:36). Reads of all samples were assembled using Trinity r20131110 [40], which yielded 249 855 and 150 314 transcripts for *E. cornigera* and *E. timida*, respectively. Expression values for transcript clusters (or unigenes) were extracted and analysed using the Trinity pipeline [41] via RSEM 1.2.11 [42], Bowtie 1.0.1 [43] and edgeR 3.4.2 [44]. Only unigenes with raw read counts of greater than or equal to 100 over at least two samples were analysed, resulting in 36 380 and 32 897 unigenes, respectively. Differential expression was determined by log_2_fc of at least ±1 compared to the control set of unigenes (with raw read counts of greater than or equal to 100). Expression change significance was assessed based upon the dispersion of available replicate information in edgeR. The longest isoform of each cluster was defined as the representative sequence for a unigene.

To plot the overall taxonomic distribution of the assembled transcripts, unigenes were subjected to a BLASTx-based [45] search (*e*-value cut-off 10^−10^) against a database consisting of protein sequences of RefSeq version 64 [46] plus those of the genomes of *Crassostrea gigas* [47] and *Lottia gigantean* [48] (electronic supplementary material, figure S2). For all downstream analyses, only those genes were included for which top hits to the mentioned organisms or other metazoans were retrieved. Excluded were those with best blast hits to plants, protists, prokaryotes and viruses. Protein annotations for the 14 848 *E. cornigera* and 13 875 *E. timida* unigenes were extracted from mentioned BLAST hits and from a second BLASTx search to the UniProtKB/Swiss-Prot database [49]. KOG/NOG (EuKaryotic/Non-supervised Orthologous Groups [50–52]) categories were determined based on the best BLASTx hit to protein sequences of eggNOG v. 4 [53]. KEGG [54] accessions were obtained using the KAAS 1.6a webservice [55] against metazoa.

For qRT-PCR, 200 ng RNA was reverse transcribed using random hexamers and the iScript Select cDNA Synthesis Kit (Bio-Rad). Two-step qRT-PCR was performed on biological triplicates for each time point and treatment (each containing pooled RNA from greater than or equal to five slugs) using the SsoAdvanced Universal SYBR Green Supermix (Bio-Rad) according to the manufacturer's instructions on a StepOne Plus (Applied Biosystems) real-time PCR system. Primers were designed using Primer-BLAST [56] (for primer sequences, see the electronic supplementary material, table S4) and data were analysed according to Pfaffl [57], using the *Ec*RPL38 (Eco000149), *Et*RPL19 (Eti000121) and *Et*SETMAR (Eti000317) as reference genes.

## Supplementary Material

Supplementary information

## Supplementary Material

Table S3

## Data accessibility

All sequencing data was deposited at the NCBI Single Read Archive (SRP047359) and the Transcriptome Shotgun Assembly Sequence Database (*E. cornigera*: GBRW00000000; *E. timida*: GBRM00000000).
