# Supplementary material for: Comparison of sister species identifies factors underpinning plastid compatibility in green sea slugs
Source: Proc Biol Sci. 2015 Mar 7;282(1802):20142519. doi: 10.1098/rspb.2014.2519 (PMC4344150; doi:10.1098/rspb.2014.2519)
Supplement: Supplementary information [file rspb20142519supp1.pdf]

Supplementary figure and table captions

Figure S1. Pigmented kleptoplasts are present in *E. cornigera* that starved to death. Exemplary fluorescence micrograph of the autofluorescent kleptoplasts in the digestive tubule of an *E. cornigera* individual that had died from starvation after 11 days.

Figure S2. Taxonomic distribution of unigenes. Classifications of filtered unigenes (14,848 for *E. cornigera* and 13,875 for *E. timida*) were inferred from their best BLAST hits. Numbers and colours indicate for the absolute amounts and classes of the different phylogenetic groups or organisms.

Figure S3. Conformation of global gene expression trends through quantitative real-time PCR. Relative expression of three genes of each, *E. cornigera* (Eco000004, Eco000150, Eco000152) and *E. timida* (Eti000009, Eti000070, Eti000123) were analysed using two-step quantitative reverse transcription PCR (qRT-PCR; top two panels) to confirm the trends obtained for these genes through the RNAseq analyses (bottom two panels). All expression data is given as a log2(fold change) obtained from the different conditions applied (S, starvation alone, S+M, starvation and Monolinuron treatment, S+B, starvation and highlight stress; number of days [d] indicates the duration of the conditions) relative to the expression data obtained from feeding slugs. qRT-PCR data was retrieved from biological triplicates (≥7 E. cornigera and ≥5 E. timida per replicate per timepoint and condition) and ΔΔct-normalized using EcRPL38 (Eco000149), EtRPL19 (Eti000121) and EtSETMAR (Eti000317) as reference genes. Error bars indicate standard deviation for the biological triplicates.

Figure S4. Feeding slugs accumulate no hydrogen peroxide. Confocal laser scanning micrographs of the kleptoplast-bearing digestive tubules that pervade the parapodia of whole mount *E. cornigera* and *E. timida* stained with 100µM 2',7'-dichlorofluorescin (DCF; blue, *a-d*) and MitoTracker Red (green, *d*); chlorophyll autofluorescence is shown in red (*a-d*). Duration of starvation is given in days. *E. cornigera* and *E. timida* accumulate no visible amount of H_2O_2 in their digestive when actively feeding (*a*, *b*). After 30d of starvation the digestive tubules of *E. timida* appear thinner, but still show no traceable amounts of H_2O_2 (*c*), but globular structures of unknown nature begin to appear that accumulate H_2O_2 (arrowhead in *c*). Details of a single digestive tubule in *E. timida* after 10d starvation showing the co-localising of MitoTracker Red with the DCF staining (*d*, arrowheads).

Table S1. Kleptoplast pigment concentrations of *E. cornigera* and *E. timida* throughout starvation. Concentrations of the six pigments are given in nmol/µg dry weight. MV, mean value; SD, standard deviation.

Table S2. Overview of the transcriptomes analysed. Nine transcriptomes of *Elysia cornigera* and 12 transcriptomes of *Elysia timida* were sequenced in total.

Table S3. Annotations and expression values of analysed *Elyisa timida* and *Elyisa cornigera* unigenes. All expression values are in FPKM (fragments per kilobase of exon per million reads mapped). S, starvation alone; S+M, starvation plus Monolinuron treatment; S+B, starvation plus 1 h high light stress of 1000 μmol quanta $m^{-2}s^{-1}$.

Table S4. Sequences for the oligonucleotides used for the quantitative reverse-transcription PCRs.

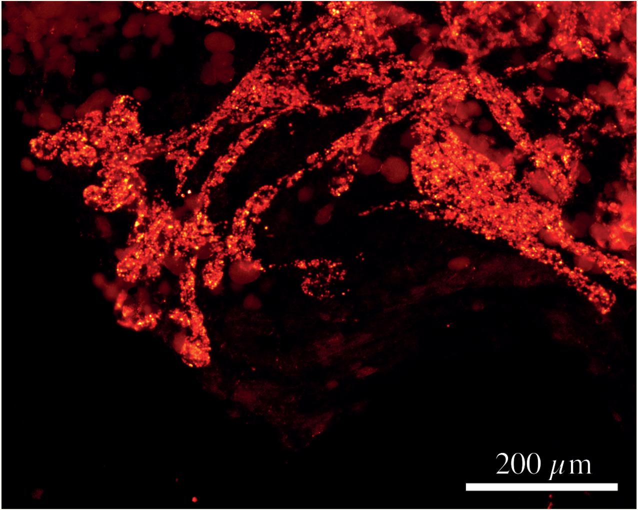
200 μm

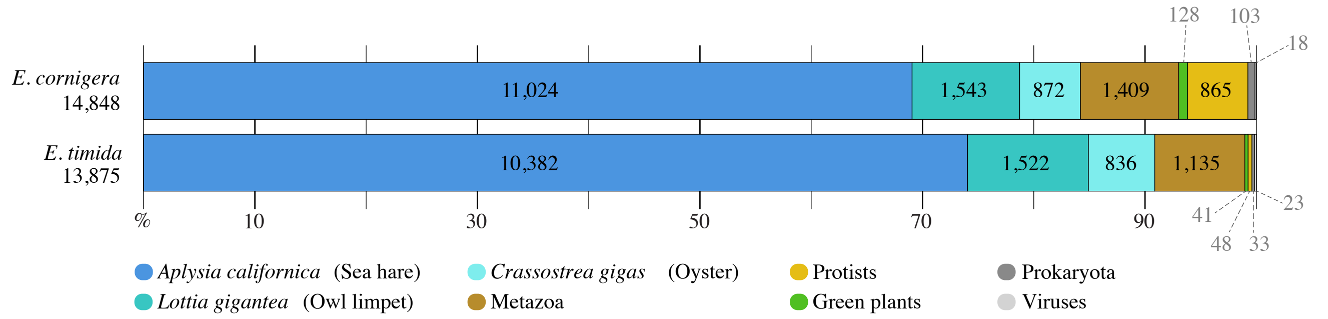

E. cornigera
14,848
E. timida
13,875
11,024
1,543
872
1,409
865
128
103
18
10,382
1,522
836
1,135
41
48
33
23
%
10
30
50
70
90
Aplysia californica (Sea hare)
Lottia gigantea (Owl limpet)
Crassostrea gigas (Oyster)
Metazoa
Protists
Green plants
Prokaryota
Viruses

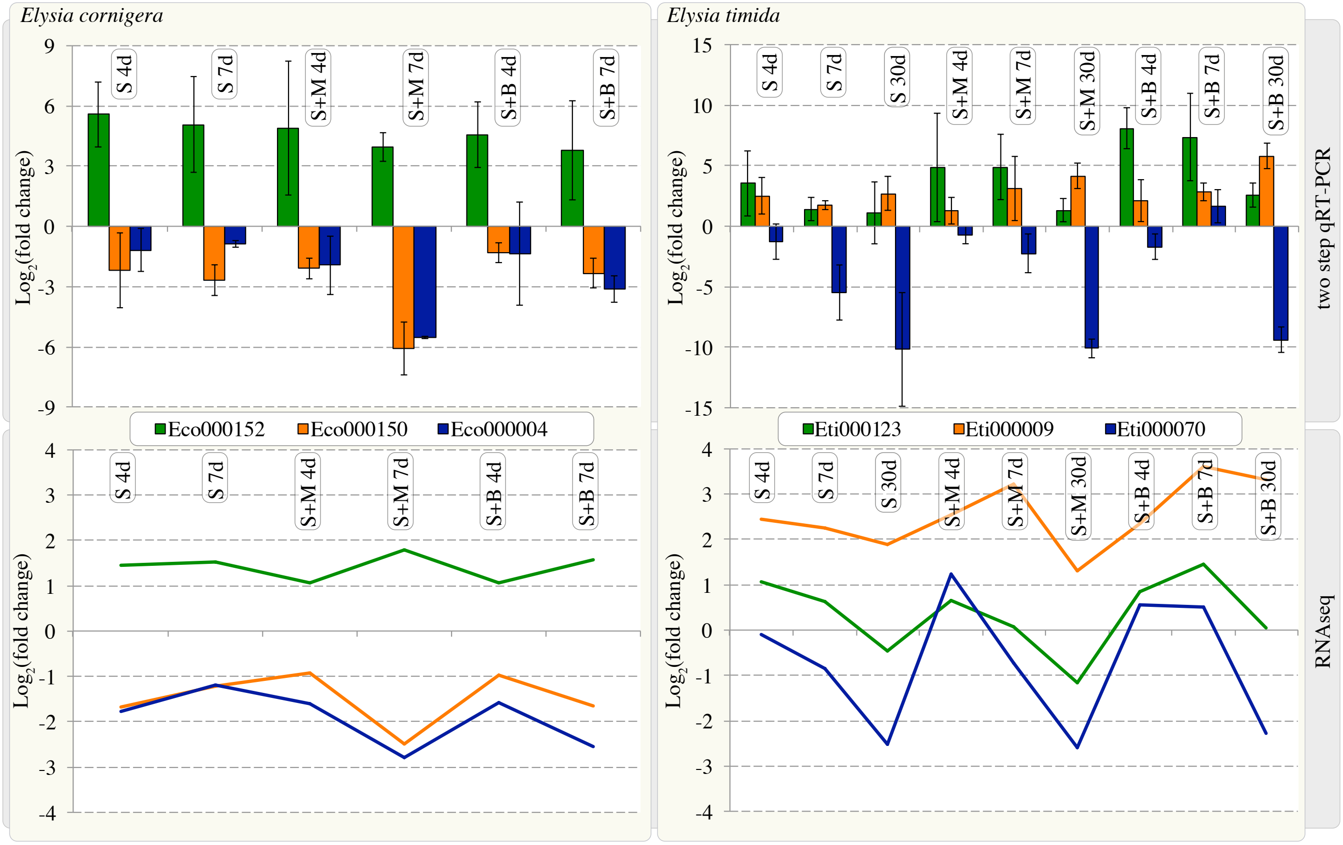

Elysia cornigera
Elysia timida
Log2(fold change)
S 4d
S 7d
S 30d
S+M 4d
S+M 7d
S+M 30d
S+B 4d
S+B 7d
S+B 30d
Eco000152
Eco000150
Eco000004
Eti000123
Eti000009
Eti000070
two step qRT-PCR
RNAseq

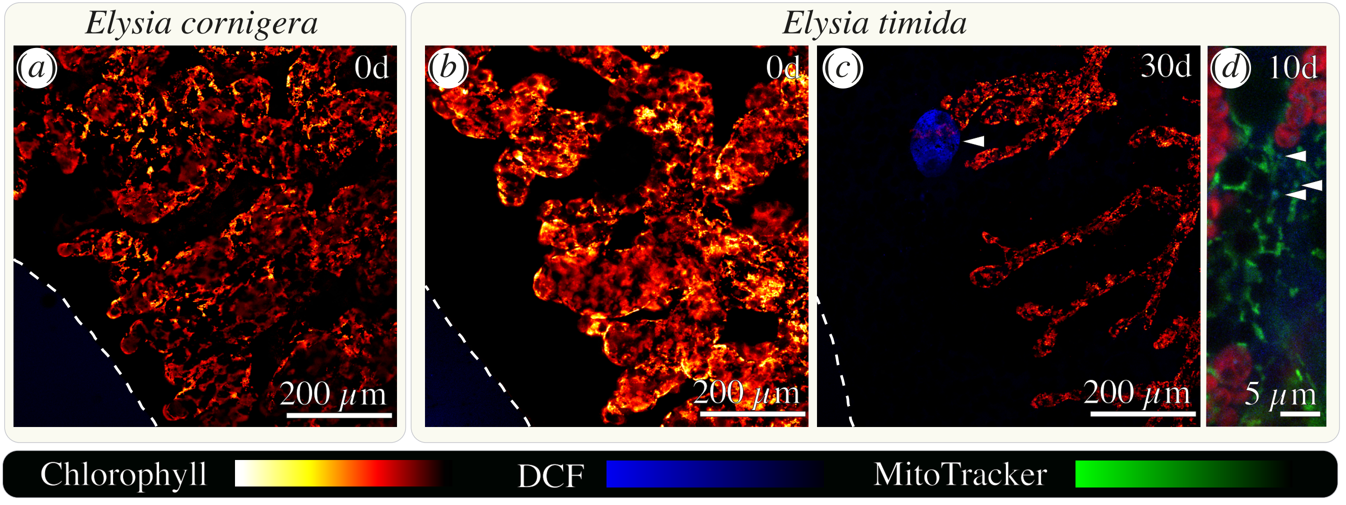

Elysia cornigera
Elysia timida
(a)
0d
200 μm
(b)
0d
200 μm
(c)
30d
200 μm
(d)
10d
5 μm
Chlorophyll
DCF
MitoTracker

Table S1: Kleptoplast pigment concentrations of *E. cornigera* and *E. timida* throughout starvation. Concentrations of the six pigments are given in nmol/µg dry weight. MV, mean value; SD, standard deviation.

	Starvation [d]	Lutein		Neoxanthin		Violaxanthin		Zeaxanthin		Chlorophyll a		Chlorophyll b		Sum Carotenoids		Sum Chlorophylls	
		MV	SD	MV	SD	MV	SD	MV	SD	MV	SD	MV	SD	MV	SD	MV	SD
Elysia cornigera	0	474.39	78.13	126.86	22.81	160.15	38.83	10.74	4.05	2205.20	201.13	903.70	154.64	772.14	138.78	3108.91	351.31
	1	724.85	205.76	188.57	65.44	238.32	83.27	17.15	6.65	2459.11	1085.20	1089.87	301.46	1168.89	351.04	3548.98	1371.18
	2	693.09	35.51	174.00	18.41	238.88	25.11	12.63	1.79	2562.19	619.72	1170.79	202.30	1118.60	75.87	3732.98	792.34
	3	567.22	216.04	142.42	45.48	186.77	86.01	11.81	2.95	2087.57	693.73	890.85	302.09	908.22	348.18	2978.41	989.00
	4	441.70	67.96	117.27	24.32	138.00	25.62	7.64	1.23	1799.01	668.22	749.59	220.85	704.62	112.53	2548.60	888.41
	5	406.66	35.56	113.58	21.83	143.12	7.71	8.15	0.65	1841.06	598.03	785.01	202.16	671.51	50.67	2626.07	800.18
	6	486.49	197.50	131.76	52.24	174.20	96.17	10.15	3.08	2129.48	568.81	904.36	303.99	802.60	348.44	3033.83	872.52
	7	536.62	135.09	217.56	173.26	292.46	277.14	22.16	13.19	2153.46	700.28	828.55	209.40	1068.80	556.04	2982.01	909.68
	8	425.01	93.15	112.91	26.56	140.00	46.72	12.29	5.09	2062.43	437.78	806.34	163.12	690.21	170.45	2868.77	599.81
	9	314.82	142.83	81.43	25.80	84.66	43.23	11.10	5.03	1533.77	613.53	563.89	191.17	492.01	216.89	2097.65	804.70
	10	217.67	57.56	62.31	13.40	59.43	16.18	7.74	1.03	1112.94	231.84	430.58	84.25	347.14	86.89	1543.52	315.27
Elysia timida	0	359.23	105.64	124.85	70.33	127.01	61.76	21.19	16.13	1351.45	722.66	561.27	575.94	632.27	221.60	2193.35	1159.56
	1	478.74	3.96	160.37	15.91	157.87	15.92	18.23	9.56	1878.61	110.07	1225.56	74.84	815.20	27.16	3104.17	184.74
	2	441.97	16.49	160.21	32.42	142.26	14.61	14.55	6.65	1673.32	490.37	1121.17	319.49	758.98	53.88	2794.49	809.86
	3	309.13	106.61	103.58	46.16	108.41	31.92	10.77	1.08	1280.20	674.06	818.65	438.43	531.88	185.39	2098.85	1112.29
	4	274.55	25.28	74.92	4.69	81.01	1.55	13.30	1.79	991.04	51.31	615.32	38.38	443.78	29.74	1606.36	89.69
	5	242.49	116.80	68.78	38.18	68.02	40.26	25.57	1.65	748.59	512.85	454.69	333.03	404.87	192.89	1203.28	845.45
	6	283.91	87.27	93.73	26.98	104.07	31.43	9.27	5.32	1195.09	358.62	713.92	268.09	490.98	147.00	1909.01	626.70
	7	335.67	101.62	75.10	20.65	83.55	25.29	23.22	5.50	1207.45	524.73	745.43	347.30	397.91	228.39	1952.87	872.03
	8	255.60	4.14	79.28	0.98	88.94	10.32	19.57	0.96	945.82	24.00	571.82	7.59	443.39	16.40	1517.64	31.59
	9	254.22	87.51	75.37	22.48	85.55	22.51	10.74	6.53	945.97	236.41	538.92	188.07	425.88	139.03	1484.89	424.48
	10	273.04	30.57	85.14	14.46	89.52	16.07	16.64	9.94	1015.25	216.48	622.95	114.52	464.33	54.09	1638.20	329.67
	30	270.76	195.40	67.14	21.65	49.49	17.62	31.63	9.31	1049.51	996.04	534.86	589.75	419.02	222.02	1584.38	1585.69

Table S2: Overview of the transcriptomes analysed. Nine transcriptomes of *E. cornigera* and 12 transcriptomes of *E. timida* were sequenced in total.

E. cornigera

	0 days			4 days			7 days			
	**Replicate A**	**Replicate B**	**Replicate C**	**Starving**	**S+M**	**S+B**	**Starving**	**S+M**	**S+B**	**Total**
Obtained reads	55.859.430	58.670.092	60.931.062	54.188.018	55.099.804	57.767.542	52.415.642	53.653.654	53.859.162	502.444.406
Filtered reads	51.679.348	53.731.218	56.177.474	49.960.804	51.029.300	53.294.008	48.382.758	49.564.714	49.576.816	463.396.440
Total bases	4.522.206.145	4.677.841.251	4.899.573.620	4.285.770.386	4.470.420.425	4.666.460.660	4.143.410.460	4.341.493.642	4.339.573.745	40.346.750.334
GC	40,20%	40,93%	40,61%	39,60%	39,90%	40,31%	40,71%	39,47%	39,97%	40,20%
Assembled transcripts	-	-	-	-	-	-	-	-	-	249.855
Assembled unigenes	-	-	-	-	-	-	-	-	-	161.426
Expression filtered unigenes	-	-	-	-	-	-	-	-	-	36.380
Metazoa filtered unigenes	-	-	-	-	-	-	-	-	-	14.848
Length of complete assembly in nt	-	-	-	-	-	-	-	-	-	38.800.821
Longest unigene length in nt	-	-	-	-	-	-	-	-	-	28.246
N50 in nt	-	-	-	-	-	-	-	-	-	3.125
Average unigene length in nt	-	-	-	-	-	-	-	-	-	2.613
GC	-	-	-	-	-	-	-	-	-	42,50%
Unigenes with >100 reads	8.202	8.698	8.591	9.184	8.778	9.240	8.766	8.053	8.784	10.857
Max. count for a single unigene	1.239.999	879.405	1.089.636	1.130.976	1.207.681	1.080.621	1.168.034	1.482.005	1.248.915	1.482.005

E. timida

	0 days			4 days			7 days			30 days			
	**Replicate A**	**Replicate B**	**Replicate C**	**Starving**	**S+M**	**S+B**	**Starving**	**S+M**	**S+B**	**Starving**	**ing + Monolinuron**	**Starving + Bleach**	**Total**
Obtained reads	53.296.446	61.554.786	61.537.690	53.989.786	54.094.776	55.961.340	56.889.734	54.108.772	53.543.872	59.263.070	57.724.284	61.996.524	683.961.080
Filtered reads	48.915.128	55.395.070	56.197.788	49.611.820	49.522.270	52.106.504	52.382.420	49.737.358	49.743.770	56.821.266	55.463.624	59.448.344	635.345.362
Total bases	4.184.893.824	4.818.712.309	4.885.396.240	4.337.789.118	4.221.326.516	4.576.080.774	4.584.130.662	4.352.300.090	4.360.214.170	5.029.762.633	4.912.917.403	5.261.492.792	55.525.016.531
GC	40,61%	42,28%	40,91%	39,98%	40,97%	39,93%	39,91%	39,87%	41,01%	39,87%	39,82%	40,05%	40,43%
Assembled transcripts	-	-	-	-	-	-	-	-	-	-	-	-	150.314
Assembled unigenes	-	-	-	-	-	-	-	-	-	-	-	-	98.752
Expression filtered unigenes	-	-	-	-	-	-	-	-	-	-	-	-	32.897
Metazoa filtered unigenes	-	-	-	-	-	-	-	-	-	-	-	-	13.875
Length of complete assembly in nt	-	-	-	-	-	-	-	-	-	-	-	-	41.490.095
Longest unigene length in nt	-	-	-	-	-	-	-	-	-	-	-	-	29.832
N50 in nt	-	-	-	-	-	-	-	-	-	-	-	-	3.629
Average unigene length in nt	-	-	-	-	-	-	-	-	-	-	-	-	2.990
GC	-	-	-	-	-	-	-	-	-	-	-	-	41,90%
Unigenes with >100 reads	8.217	8.359	8.620	7.964	7.639	7.953	9.126	8.585	6.827	10.276	10.201	9.978	11.152
Max. count for a single unigene	649.381	1.907.753	1.184.397	913.099	1.218.356	981.278	623.976	822.058	959.513	350.544	409.367	550.039	1.907.753

[a]Reads were preprocessed by the sequencing company

[b]Read count of at least 100 over two samples

[c]Best BLAST hit in metazoa (

Table S4: Sequences for the oligonucleotides used for the quantitative reverse-transcription PCRs.

Target	Forward	Reverse
Eco000004	AACAGGGTGGAGTGAAGTCT	TGGCAGTTGTGTCATACGCT
Eco000149	TCCAACCATCTCCTGTGCTC	GGCTGCATCGAACCTTGAAC
Eco000150	CGCTCGCTACGCACATTTC	CAGTTCCTCGTGTTGTCGGG
Eco000152	CGGCTGTTGTAGCGTGTCT	ACTGTCTTCCCAGAGTCCCG
Eti000009	TTGGGAGACGCAAGGAAAGG	ATCAAACAGGGAGGAGACGG
Eti000070	TGTTGATACGCAGAGGTGGC	ACCGTGATGAGAGAGAGTGGA
Eti000121	TCTGCCTTCTTCTTGTGAATGGA	GCCAATGCTCGTATGCCTGT
Eti000123	AGCGAGTAGACCAGAACGC	GGGCAAACAGAGTGTAGGGT
Eti000317	AAACTGAAAGCGAGGTTGCG	ATAAGGCGGGTGCGGTAATG
